# Tocilizumab and Active Antibody-Mediated Rejection in Kidney Transplantation: A Literature Review

**DOI:** 10.3389/fimmu.2022.839380

**Published:** 2022-04-14

**Authors:** Lara Cabezas, Thomas Jouve, Paolo Malvezzi, Benedicte Janbon, Diane Giovannini, Lionel Rostaing, Johan Noble

**Affiliations:** ^1^ Nephrology, Hemodialysis, Apheresis and Kidney Transplantation Department, University Hospital Grenoble, Grenoble, France; ^2^ University Grenoble Alpes, Grenoble, France; ^3^ Pathology Department, University Hospital Grenoble, Grenoble, France

**Keywords:** kidney transplantation, tocilizumab, antibody-mediated rejection, chronic active antibody-mediated rejection, estimated glomerular filtration rate, donor-specific alloantibody

## Abstract

**Introduction:**

Chronic kidney disease (CKD) is a major public-health problem that increases the risk of end-stage kidney disease (ESKD), cardiovascular diseases, and other complications. Kidney transplantation is a renal-replacement therapy that offers better survival compared to dialysis. Antibody-mediated rejection (ABMR) is a significant complication following kidney transplantation: it contributes to both short- and long-term injury. The standard-of-care (SOC) therapy combines plasmapheresis and Intravenous Immunoglobulins (IVIg) with or without steroids, with or without rituximab: however, despite this combined treatment, ABMR remains the main cause of graft loss. IL-6 is a key cytokine: it regulates inflammation, and the development, maturation, and activation of T cells, B cells, and plasma cells. Tocilizumab (TCZ) is the main humanized monoclonal aimed at IL-6R and appears to be a safe and possible strategy to manage ABMR in sensitized recipients. We conducted a literature review to assess the place of the anti-IL-6R monoclonal antibody TCZ within ABMR protocols.

**Materials and Methods:**

We systematically reviewed the PubMed literature and reviewed six studies that included 117 patients and collected data on the utilization of TCZ to treat ABMR.

**Results:**

Most studies report a significant reduction in levels of Donor Specific Antibodies (DSAs) and reduced inflammation and microvascular lesions (as found in biopsies). Stabilization of the renal function was observed. Adverse events were light to moderate, and mortality was not linked with TCZ treatment. The main side effect noted was infection, but infections did not occur more frequently in patients receiving TCZ as compared to those receiving SOC therapy.

**Conclusion:**

TCZ may be an alternative to SOC for ABMR kidney-transplant patients, either as a first-line treatment or after failure of SOC. Further randomized and controlled studies are needed to support these results.

## Introduction

Chronic kidney disease (CKD) is a major public-health problem: it increases the risk of end-stage kidney disease (ESKD) and cardiovascular disease, plus other complications. The prevalence of CKD is 10.5–13.1% when defined by the presence of albuminuria and decreased estimated glomerular filtration rate (eGFR) (1). In a meta-analysis, Hill et al. (2) reviewed 100 studies that included a total of 6,908,440 participants. Mean CKD prevalence was 13.4% (11.7–15.1%) for stages 1 and 2, and 10.6% (9.2–12.2%) for stages 3 to 5. Liyanage et al. (3) showed that, of the 2.618 million people that had received renal-replacement therapy worldwide in 2010, 2.05 million received dialysis therapy and the others received a renal transplant. Worldwide prevalence of the use of renal-replacement therapy is projected to double by 2030, up to 5 millions of patients.

It is recognized that kidney transplantation, as compared to dialysis therapy, offers better patient survival for patients with ESKD (4). In addition, kidney transplantation offers better quality of life and better cost-effectiveness as compared to dialysis (5). Progress in kidney transplantation has improved patient and graft survival: however, issues with adequate pretransplant assessment, judicious use of potent immunosuppressive therapies and management of post-transplantation complications still remain (6).

Antibody-mediated rejection (ABMR) is a significant complication following kidney transplantation that contributes towards short- and long-term injury in ~1–10% of kidney-transplant recipients and increases the risk of graft loss (7). It is characterized by allograft dysfunction, morphologic evidence of acute and/or chronic allograft microcirculation injury (glomerulitis and peritubular capilaritis) with C4d deposition in peritubular capillaries, together with the presence of circulating donor-specific anti-HLA alloantibodies (DSAs) (8).

Recently, Mayrdorfer et al. reported on a single-center study that examined the causes of allograft loss in 1642 kidney-transplant recipients. They found that 51.2% of patients had more than one cause contributing to allograft loss. The most frequent primary or secondary causes leading to graft failure were intercurrent medical events in 36.3%, T cell-mediated rejection (TCMR) in 34%, and ABMR in 30.7%. In 77.9%, a primary cause could be attributed to graft loss, of which ABMR was the most frequent (21.5%) (9).

Sellarés et al. prospectively studied 315 kidney-transplant recipients that underwent indication biopsies between 6 days and 32 years posttransplant; of these 60 patients progressed to allograft failure in the follow-up period (median 31.4 months). They reported that most graft failures manifested a phenotypic feature of ABMR or mixed rejection, and also underscored the major role of patient’s non-adherence (10).

Up to one-third of highly sensitized recipients may develop acute ABMR (aABMR) following transplantation, even in those that have undergone a pretransplant desensitization protocol (7). ABMR is also of significant concern in non-sensitized individuals as *de novo* DSAs can develop early or late after transplantation from either nonadherence or immunosuppression-minimization protocols. Thus, ABMR is seen in up to 5% of first kidney-transplant recipients (7).

Treating ABMR remains a big challenge, especially for chronic active ABMR (caABMR). The current strategy for treating aABMR is to use of a combination of therapies to target multiple pathophysiological pathways. The current standard-of-care (SOC) combines plasmapheresis and Intravenous Immunoglobulins (IVIg), with or without steroids (11–13). High-dose IVIg is often used in combination with anti-CD20 (rituximab), despite mixed evidence for its added benefit (14). In the setting of acute ABMR, the guidelines on “Kidney Disease: Improving Global Outcomes” recommend using of one or more of the following, with or without corticosteroids: plasma exchange, IVIg, anti-CD20 antibody or lymphocyte-depleting antibodies (15).

Conversely, in the setting of chronic caABMR, there are still left very few therapeutic options. caABMR was well-defined in the last Banff classification report (16). Eskandary et al. showed, in a randomized controlled trial that two cycles of bortezomib (a proteasome inhibitor) was no more efficient than a placebo at preventing a decline in eGFR, at improving histologic or molecular disease features, or at reducing DSAs, despite significant toxicity (17). However, very recently, the same group reported on a phase II randomized pilot trial that evaluated the safety (primary endpoint) and efficacy (secondary endpoint analysis) of the anti-Interleukin (IL)-6 antibody clazakizumab (vs. placebo) in 20 kidney-transplant recipients with DSA-positive ABMR at ≥365 days post-transplantation. They reported the potentially beneficial effect of clazakizumab on ABMR activity and progression (18).

**Figure 1:**
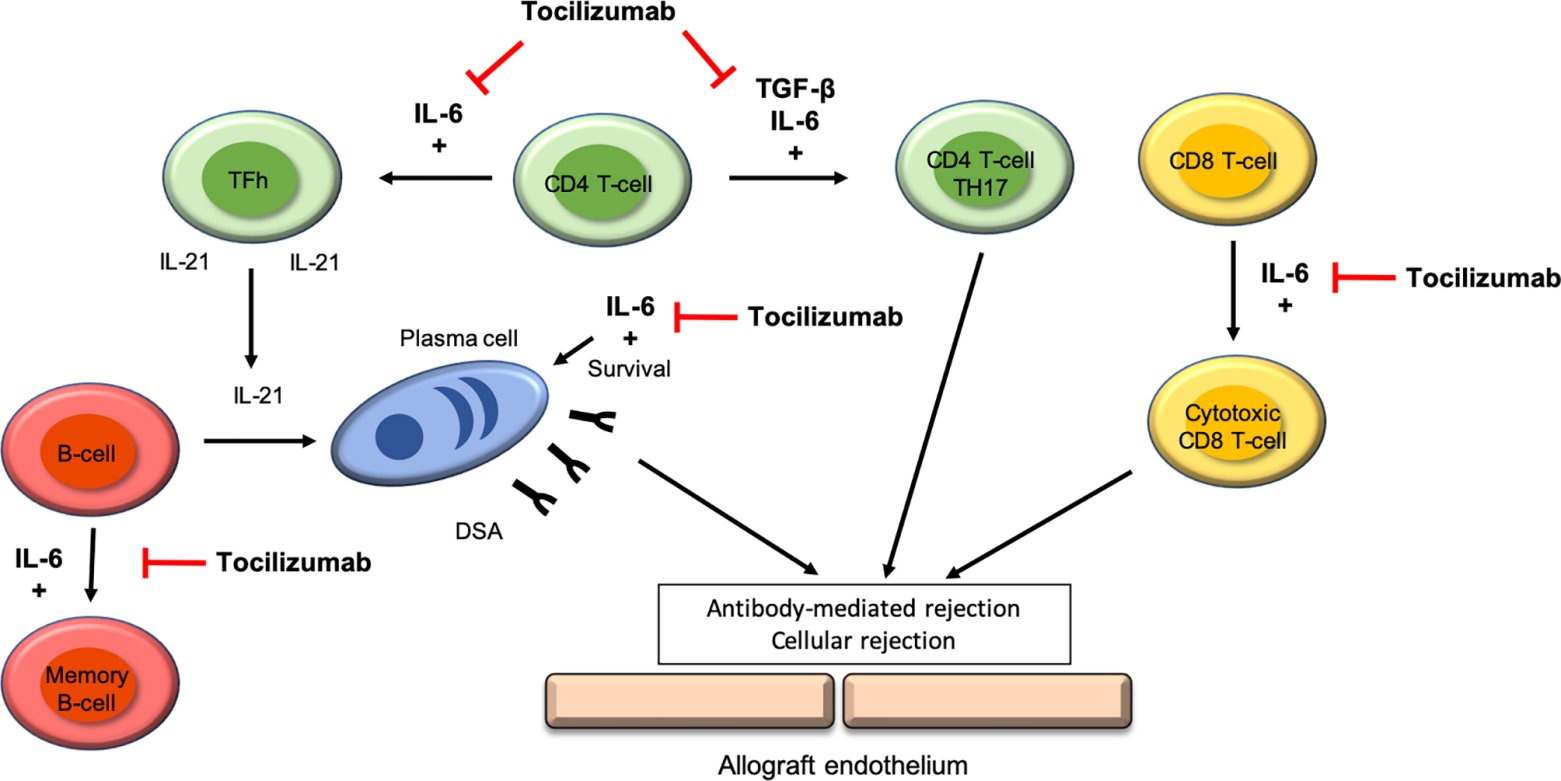
IL-6 receptor targets of Tocilizumab in the development if antibody-mediated rejection.

IL-6 is a key cytokine that regulates inflammation and the development, maturation, and activation of T cells, B cells, and plasma cells (19). Some human diseases are linked to excessive IL-6 production, characterized by unregulated antibody production and autoimmunity (19). It has been shown that the interaction between IL-6 and the IL-6 receptor (IL6-R) is part of alloantibody generation by stimulating B cells. If we block this interaction, it leads to significant reduction in alloantibodies, antibody production by splenic and bone-marrow plasma cells, direct inhibition of plasma cell anti-HLA antibody production, and induction of T regulatory cells (Tregs) with inhibition of T follicular helper cells (Tfh) (20, 21). **Figure 1** summarize the interaction of IL-6 and allograft rejection and the potential benefit of Tocilizumab in the setting of ABMR.

Tocilizumab (TCZ) is the first-in-class humanized monoclonal aimed at IL-6R: it binds to both soluble and membrane-bound forms of IL-6R. The efficacy of TCZ was first confirmed in a clinical trial involving 28 patients with Castleman disease (22) and it is now approved by the FDA for treatment of rheumatoid arthritis and juvenile idiopathic arthritis. A phase I/II trial reported that the addition of TCZ to IVIg as a desensitization therapy appeared to be a safe and feasible strategy to manage the most challenging highly HLA-sensitized kidney transplant candidates that are resistant to SOC therapy, by reducing anti-HLA antibodies and improving access to transplantation. Thus, ten kidney transplant candidates unresponsive to desensitization with IVIg + rituximab were treated with IVIg + TCZ. DSA strength and number were reduced by TCZ; in addition, five cases could then receive a transplant. Protocol kidney-allograft biopsies at 6 months post-transplantation showed no ABMR (23).

Based on these findings, TCZ may have a positive impact in ABMR, by reducing inflammation, alloantibody titers, and by targeting many pathways within the immune response involved in ABMR. Very recently, we and other groups have studied TCZ in the setting of ABMR in kidney-transplant recipients. This prompted us to carry out a literature review to evaluate the efficacy and safety of the anti-IL-6R monoclonal antibody TCZ in the setting of caABMR. Herein, we discuss TCZ’s place in caABMR therapeutic strategies.

## Materials and Methods

We performed a systematic review of the literature using the PubMed search engine with the following MeSH terms: kidney transplantation; antibody-mediated rejection; late antibody-mediated rejection; chronic active antibody-mediated rejection; tocilizumab.

## Results

### The Studies’ Characteristics

In **Table 1**, we collected characteristics from the studies’ populations. We included six studies: three studies in which TCZ was used as a rescue therapy for caABMR, two studies in which TCZ was used as a first-line therapy to treat caABMR and one study that in which TCZ was used as a rescue therapy for aABMR.

**Table 1:** Study characteristics.

Study	Number of subjects Design	Age (yrs)	Second transplantation or more, No (%)	Acute/ChronicActive Antibody-mediated rejection	Anti-HLA DSA + at the time of AMR, No (%)	Histology	Renal function at baseline	Follow-up
Choi et al. (24) *American Journal of Transplantation* 2017	36 patients single-center, open-label case study	Mean (SD) 45.86 (16.64)	17/36 (47.2)	Chronic	33/37 (91,67)	Mean g score : 1.67 ± 1.11 ptc score : 1.78 ± 0.58 C4d : 1.54 ± 1.50	Mean eGFR (SD) 48.43 mL/min/1.73 m2 (34.56)	Median (SD) 3.26 (2.04)
Massat et al. (25) *American Journal of Transplantation* 2021	46 Patients in 2 groups - 9 SOC + tocilizumab - 37 SOC Retrospective study	Mean (SD) SOC+TCZ : 37.2 (14.1)*	SOC+TCZ 4/9 (44.4)*	SOC + TCZ 2/9 mixed rejection 6/9 chronic active rejection*	SOC+TCZ 6/9 (66.7) SOC 12/37 (32.4)	Mean g+ptc score -SOC+TCZ 3.0 ± 0.82*	SOC + Tocilizumab Median eGFR (min-max) 40 mL/min/1.73m² (25-55)*	1 year post AMR rdiagnosis
Lavacca et al. (26) *Clinical Transplantation* 2020	15 patients single‐center, open‐label, non-sponsored case study	Median (SD) 38.3 (23.0-47.5)	2/15 (13,3)	Chronic	15/15 (100)^ⱡ^	Median (SD) cg score 3 (2-3) g + ptc score 3 (2-4)	Median proteinuria 1.1 g/day Median eGFR (SD) 54.5 mL/min/1.73m² (47.5-56.8)	Median 20.7 months after first TCZ
Pottebaum et al. (27) *Transplantation Direct* 2020	7 patients Case report	Mean 42.9	4/7 (57,1)	Acute	7/7 (100)	3/7 C4d staining 4/7 ptc3, 2/7 ptc 2 and 1/7 ptc 0 2/7 g0, 4/7 g1, 1/7 g2	NA	Inclusion between October 2016 and October 2018, followed through august 2019
Kumar et al. (28) *American Society of Nephrology* 2020	10 patients Retrospective study	Mean (SD) 43 (34.6-51.4)	4/10 (40)	Chronic	8/10 (80)	Mean g+ptc score 4.8 ± 1.4	Mean eGFR 42 ± 19ml/min/1.73m2 Mean proteinuria 1.6 ± 1.1g/g	Median 12 months (range=8-24)
Noble et al. (29)	40 patients single-center study	Mean (SD) 43 ± 15	10/40 (25)	Chronic	22/40 (55)	50% had g ≥2 and ptc ≥2 25% had cg ≥ 2 18% had IFTA ≥ 2	Mean eGFR 43 ± 17 mL/min/1.73m2 mean proteinuria 1.0 ± 0.9 g/L	Median 7 (4—13) months

NA, Non assessed.

*There were no significant differences between the SOC group and the SOC+TCZ group.

ⱡMostly anti class II or de novo.

There was a total of 117 TCZ-treated kidney transplant recipients. One of the six studies included a control group, i.e., a historical cohort. Three of the studies were single-center open-label. All studies assessed patient- and graft-survival rates, as well as renal function (eGFR) and DSA outcomes. Histology before and after treatment was assessed in all studies except in the case reports where patients only had a biopsy at the time of ABMR diagnosis. Adverse events and tolerability were noted in all studies except for one. Patients were followed for a median period of one-year post-TCZ therapy and most patients were male. Immunosuppressive treatments at baseline were very similar in all studies: i.e., induction included anti-thymocyte globulins (or basiliximab for one study) and a maintenance therapy of calcineurin inhibitors (mostly tacrolimus), an anti-metabolite (mostly mycophenolic acid), and steroids. TCZ was used at a dose of 8 mg/kg monthly with a maximal dose of 800 mg.

### Efficacy of Tocilizumab for Resistant Chronic Antibody-Mediated Rejection

In three studies, TCZ was given for resistant to SOC caABMR. The overall main results are shown in **Table 2**.

**Table 2:** Outcomes.

Study	Renal function	Graft survival	Patient survival	DSA	Histology
Choi et al. (24) *American Journal of Transplantation* 2017	eGFR stable	80% at 6 years post–cAMR Diagnosis Graft loss 4/36 (11%)	91% at 6 years post-AMR diagnosis	Reduction on the immunodominant DSA at 24 months (p = 0,043)	Reduction in g+ptc score (p = 0.0175) Reduction of C4d+ (p = 0.0318) one year after TCZ treatment
Massat et al. (25) *American Journal of Transplantation* 2017	Decline -4.0 ml/min/1,73m²/year (SOC+TCZ)*	6/9 in the tocilizumab group One year graft survival similar in the two groups	100% at 1 year follow-up	Overall decrease in the MFI of DSA with TCZ Variation in MFI : - 48 ± 44% SOC+TCZ+107% ± 94% SOC (p= 0.01)	inflammation and tubulitis : - decrease 1.78 ± 1.40 to 0.57 ± 0.79, p = 0.07 (SOC+TCZ) - stable 0.75 ± 1.25 to 1.0 ± 1.30, p = 0.47 (SOC) no significant in active antibody-mediated lesions or chronic glomerulopathy No difference in the C4d deposition
Lavacca et al. (26) *Clinical Transplantation* 2020	eGFR and 24-hour proteinuria : stabilization at the 12 month follow-up eGFR declined by 10.5 ml/min/1.73m2 (median) in the 12 months before cAMR diagnosis compared to 4.4 ml/min/1.73m2 the first year after diagnosis.	14/15 Graft loss : 1/15 (6,7), 30 months after diagnosis and 25,3 months after initiation of TCZ treatment	100%	Mean MFI declined 22600, 21700-23700 pre-TCZ 18200, 12650-22150 post-TCZ (p = 0.002)	Decrease in microvascular inflammation (g + ptc score) 3 (2-4) pre-TCZ 2 (1-2.5) post-TCZ (p = 0.014) absence of progression in chronicity scores (cg and IF/TA) or C4d deposition
Pottebaum et al. (27) *Transplantation Direct* 2020	Stable renal function in 4/7 patient	5/7 with stable renal function except one with T-cell mediated rejection after 24 months with noncompliance Graft loss : 2/7 graft loss -one with T-cell mediated rejection after 18 months with return to dialysis -one with mixed rejection after 6 months with return to dialysis	100%	3/7 decline of the MFI of the iDSA 1/7 negative 2/7 stable 1/7 NA	NA
Kumar et al. (28) *American Society of Nephrology* 2020	eGFR stabilization (p = 0.428) proteinuria stabilization (p = 0.697)	overall death-censored graft survival 80%	90%, with one patient death due to complications from a post-surgical hip infection with a functioning graft	Mean total DSA unchanged 7272 ± 6698 MFI pre-TCZ 6273 ± 8480 MFI post –TCZ (p = 0.629)	no improvement in histologic mean microvascular inflammation (g+ptc) scores (p = 0.394) numerical worsening of histologic interstitial fibrosis and tubular atrophy (CI+CT) scores (p = 0.383) as well as overall chronicity (CI+CT+CG+CV) scores (p = 0.286) without significant difference
Noble et al. (29)	eGFR stable at 6 months (p = 0.12) and 12 months (p = 0.102) proteinuria stabilization at 6 months (p = 0.95) and 12 months (p = 0.28)	85% at one year 6 graft loss which have lower eGFR, more sever histological presentation	100%	NA	no statistical difference in the follow-up histologic scores except for the intimal arteritis score

NA, not assessed.

*There were no significant differences between the SOC group and the SOC+TCZ group (p = 0.18).

Choi et al. (24) were one of the first team to study the anti-IL6R monoclonal antibody as a treatment for caABMR. TCZ was used as a rescue therapy for patients with DSA positive biopsy proven caABMR. Thirty-six patients were enrolled: most had a progressive CKD and had failed the first line treatments, i.e. IVIg plus rituximab, with or without plasmapheresis. They were monitored for DSAs every three months and eGFR was assessed every month for up to 6 years. Nine patients underwent follow-up biopsies at 1-year post-TCZ treatment to assess pathological features. Graft- and patient-survival rates were excellent: respectively, 80% and 91% at 6 years post-caABMR diagnosis. Only four graft losses were observed due to caABMR, occurring in patients with TCZ premature discontinuation. This might suggest a rebound effect related to the accumulation of IL-6 during TCZ inhibition of IL-6R.

Renal function was stable during the study. They also showed a significant decrease in immunodominant DSA (iDSA). iDSA refers to the DSA with the highest mean fluorescence intensity (MFI) in a serum sample and iDSA MFI is well correlated with AMR occurrence. In biopsies, a significant reduction in C4d+ deposition and in the microvascular circulation ‘g plus ptc’ score were seen post-TCZ treatment. Those pathological features have been shown to represent the most relevant signature of ABMR and are associated with worse long-term outcomes (30). The most frequent adverse events were bacterial or viral infections. The two reported deaths were described to be unrelated to TCZ treatment.

Massat et al. (25) reported different results. They performed a retrospective study on kidney transplanted patients with a biopsy-proven DSA positive ABMR, resistant to SOC therapy. They compared two groups: nine patients that received TCZ in addition to SOC therapy and 37 patients from a historical cohort that only received SOC therapy. At TCZ initiation for the 9 included patients, six patients presented with caABMR, three with TCMR. Four patients had mixed ABMR/TCMR features. During the one-year follow-up, three patients from the TCZ group lost their graft due to caABMR. Nevertheless, the one-year graft-survival rate at post-diagnosis of resistant ABMR was similar between the two groups. There were no differences in the eGFR decline. They also found a significant reduction in MFI of anti-class-I and anti-class-II HLA antibodies in the TCZ group as compared to the SOC group. The pathological follow-up showed that, despite a decrease in interstitial inflammation (i) and tubulitis (t) scores after TCZ, the course of antibody-mediated lesions and chronic glomerulopathy were similar in both groups. Those results differed from the results of Choi et al. The main side effect was non-lethal infection complications.

Another study reported relatively similar results. Kumar et al. (28) treated ten patients that had refractory caABMR with TCZ and compared pre- and post-TCZ biopsies. Patient survival was 90% at one year. One patient death was caused by a post-surgical hip infection and the kidney graft was functional. There were two graft losses: these two patients had recurrent infections that required hospitalizations and TCZ discontinuation. There was no worsening in renal function throughout the follow up period: mean eGFR was 42 ± 19 mL/min/1.73 m^2^ pre-TCZ versus 37.2 ± 19 mL/min/1.73 m^2^ at 6 months (*p*=0.428) and 37 ± 24 mL/min/1.73 m^2^ at the last follow-up (*p*=0.274). This study also showed no worsening in the microvascular inflammation scores (g plus ptc): 4.2 ± 2.0 versus 4.8 ± 1.4; *p*=ns. However, chronicity score worsened without reaching a statistical significance. Mean total DSAs MFI at the time of therapy was 7272 ± 6698 and remained unchanged post-TCZ: 6273 ± 8480 (*p*=ns).

The differences between this study and Choi et al. may be explained by the fact that the population had almost a two-fold higher degree of microvascular inflammation with a mean g+ptc score of 4.10 ± 1.52 compared to 1.67 ± 1.11 in the study of Choi et al., and a three-fold higher degree of interstitial fibrosis/tubular atrophy (IFTA) with a mean ci+ct score of 2.80 ± 0.79 compared to 0.93 ± 0.72. Another relevant difference between these two studies was the use of belatacept in seven of ten patients in Kumar et al. study. These results should be considered with caution because of the small number of patients and the absence of control group.

A more recent single-center trial was conducted by Noble et al. (29) to assess long-term effects of monthly therapy of TCZ given to kidney-transplant recipients presenting with caABMR. Forty patients were enrolled between August 2018 and July 2021. Median time to caABMR diagnosis was 18.9 [6 – 55] months post-kidney transplantation. Regarding to kidney function: eGFR remained stable between baseline and 12-months follow-up, assessed using a linear regression model; however, if patients that lost their allograft (n=6) were taken into account (as eGFR = 5 ml/min/1.73m2), there was a slight decline in eGFR at last follow-up. Proteinuria remained stable at 12-months. Six patients lost their graft. We compared to those that did not lose their graft, they had significantly lower eGFR at baseline, a higher proteinuria output, and more severe histological lesions. Noble et al. then compared Banff scores from paired kidney biopsies between baseline and first-year follow-up post-TCZ treatment in 20 matched patients. There were no statistical differences in the acute and chronic histologic scores at 1-year follow-up. These results are in contradiction with those included in this review (24–26). DSA levels at 6 and 12 months were not assessed. The immunosuppressive treatment associated with TCZ was Rituximab (40%), plasmapheresis (20%), antithymoglobulin (5%) and high dose of steroids (52.5%). Seven patients received TCZ as a first-line therapy of caABMR. It is also important to note that, before TCZ treatment, one patient was receiving belatacept for maintenance anti-rejection treatment, and 18 patients were converted from calcineurin inhibitors to belatacept-based therapy after caABMR diagnostic.

### Efficacy of Tocilizumab as a First-Line Therapy in Antibody-Mediated Rejection

In order to study the efficacy of TCZ as a first-line therapy for caABMR, Lavacca et al. (26) enrolled 15 caABMR kidney transplant recipients with a severe transplant glomerulopathy and without any previous therapy, including rituximab, IVIg, plasmapheresis, high dose steroids, bortezomib, anti-thymoglobulin or complement blockers. All were treated with TCZ at a dose of 8 mg/kg (maximum of 800 mg). The median time of follow-up after the first TCZ administration was 20.7 months. They showed encouraging results with only one graft loss observed (6.7%) at 30 months after diagnosis and 25.3 months after TCZ initiation. Stabilization of eGFR and 24-hour proteinuria was reported. As was seen in Choi et al. study (24), there was a significant reduction in microvascular inflammation with a ‘g plus ptc’ score of 3 (2–4) at pre-TCZ versus 2 (1—2.5) at post-TCZ (*p*=0.014). There was no statistical difference in the progression of chronicity and C4d-deposition scores. In two cases with interstitial inflammatory edema, a remarkable decrease in inflammation post-TCZ treatment was observed in the follow-up kidney biopsy. Mean MFI values significantly declined after TCZ treatment: 22,600 [21,700—23,700] at pre-TCZ versus 18,200 [12,650—22,150] at post-TCZ; *p* = 0.002. Adverse events were mostly bacterial infections: four urinary tract infections and one pulmonary infection. Four patients developed hypogammaglobulinemia and three patients had asymptomatic mild alterations in liver enzymes. No hospitalization or TCZ discontinuation was needed.

In the retrospective study of Pottebaum et al. (27), seven patients that received TCZ to treat aABMR, in addition to conventional therapies were assessed. All patients had a renal-allograft biopsy at the time of diagnosis and DSA testing. This study showed a 50% decrease in the iDSA in four of the seven patients and a DSA stabilization in two of the seven patients, whereas it has been shown that conventional therapy with plasmapheresis followed by IVIg reduce DSA levels by 15%–35% depending on the specificity and number of plasmapheresis sessions (31). The adverse events reported included cytomegalovirus (CMV)-related esophagitis and an hypersensitivity-like reaction post injection. The authors reported two graft losses, but it should be noted that all patients included had received ≤3 doses of TCZ. We may emphasize that a longer duration of TCZ treatment may more effective.

### Adverse Events

Adverse events are summarized in **Table 3**. They were mostly infectious complications (bacterial, viral, fungal). Only one study reported a cardiovascular event in two patients: non-ST-segment elevated myocardial infarctions and one stroke. However the causal relationship with TCZ therapy in not clear. Another frequent adverse event was hypogammaglobulinemia, which may be expected due to TCZ’s effect on B-cells and plasma cells. However, hypogammaglobulinemia is also a well-known side effect of Rituximab. Although the mechanism of hypogammaglobulinemia is not well described, the effect of IL-6 on B cells proliferation and immunoglobulin class switch may be the reason. In Choi et al. (24) study, eight patients had hypogammaglobulinemia, whereas 13 patients suffered from infections, which confirms that the TCZ also impair the immune system in another way than by acting on the plasma-cells. There is a single report of hypersensitivity-like reaction post injection. Finally, TCZ therapy doesn’t seems to cause more adverse effects than other immunosuppressive therapies (25). These side-effects have to be put into perspective regarding the use of TCZ in other conditions, such as in rheumatology patients. A recent review (32) reported adverse events in patients with rheumatoid arthritis and treated with IV or subcutaneous TCZ, with or without other immunomodulatory treatments. TCZ is well tolerated in the clinical trial and clinical practice settings with a follow-up >9 years. Very common adverse reactions (incidence ≥10%) were upper respiratory-tract infection, which is similar to our results, and hypercholesterolemia. The incidence of infection was between 4 and 5 events/100 person years (PYs) exposure to TCZ in the ACT-UP trial (33) and 4.7/100 PYs exposure in the REGATE registry (34). In systemic sclerosis (35), there were no significant differences in disability, fatigue, itching, or in clinical global-disease severity assessment in patients from the TCZ group versus a control group, although serious infections were more common in the TCZ group: seven of 43 patients (16%) compared to the placebo group: two of 44 (4%), and one patient died in relation to TCZ treatment. Other adverse events have been reported, such as gastrointestinal perforation or elevated liver-enzyme levels, but there were much less common (55% ± 8 infectious complication versus 23% ± 3 noninfectious complications).

**Table 3:** Adverse events.

Study	Infection	Cardiovascular	Others	Death
Choi et al. (24) *American Journal of Transplantation* 2017	13/36 5 CMV 3 BK virus 7 bacterial infections	3/36 1 stroke 2 NSTEMI	1/36 transient visual disturbance 8/36 hypogammaglobulinemia	2/36 1 diabetic coma 1 bacterial pneumonia
Massat et al. (25) *American Journal of Transplantation* 2017	6/9 (SOC+TCZ)* 2 bacterial infections 2 viral infections 2 fungal infections	NA	NA	None
Lavacca et al. (26) *Clinical Transplantation* 2020	5/15 bacterial infection	NA	1 encephalitis of undefined origin 2 interstitial lung disease 4 hypogammaglobulinemia 3 asymptomatic mild alterations in liver enzymes	None
Pottebaum et al. (27) *Transplantation Direct* 2020	1/7 CMV esophagitis	NA	1 potential hypersensitivity reaction	None
Kumar et al. (28) *American Society of Nephrology* 2020	4/10 3 bacterial infection 1 viral with HSV	NA	5 leukopenia 1 severe diarrhea	1/10 due to infectious complications after hip replacement
Noble et al. (29)	NA	NA	NA	NA

NA, not assessed.

*There were no significant differences between the SOC group and the SOC+TCZ group.

## Discussion

The aim of this study was to discuss the place of the IL-6R blockers, as TCZ, in the treatment of caABMR. The main reported effect of TCZ is the significant reduction in DSA levels, and possibly a reduction in total inflammation and microvascular inflammation scores, seen in follow-up kidney biopsies. Indeed, three of the six studies showed a reduction in the ‘g plus ptc’ scores. Graft and patient survival were excellent in most studies; renal function stabilized in five studies, i.e. a significant decline in renal function was only seen in one study, but without a significant difference when compared to the historical SOC-therapy group. Adverse events were light to moderate and deaths were not linked to TCZ treatment. The most frequent side-effects were infections, but they did not appear to be more frequent in patients receiving TCZ than in those that received the SOC therapy. These results, although not randomized, are promising for the use of TCZ in resistant or as a first-line therapy for ABMR. These results need to be confirmed in large randomized prospective trials.

This Review has some limitations. Of the six included studies, only one was controlled and none were randomized. The studies were, for the most part, retrospective and patient numbers were small due to the low use of TCZ for ABMR. The follow-up times were, for the most part, 1 year, which remains quite short when studying caABMR rejection.

IL-6 is a pleiotropic cytokine composed of 184 amino acids: it was first identified as a B-cell stimulatory factor 2 that promoted immunoglobulin synthesis by activated B cells (21). IL-6 is not expressed in healthy individuals but is rapidly synthesized when infections or tissue injuries occur. IL-6 induces the differentiation of activated B cells into immunoglobulin-producing plasma cells and acts as a growth factor for hematopoiesis. In addition to B cells, IL-6 also affects T cells by inducing the specific differentiation of naive CD4^+^ T cells into effector T-cell subsets. In combination with TGF-β, it preferentially induces the differentiation of naive CD4^+^ T cells into Th17 cells. The pathogen-specific effector Th17 cells eliminate extracellular pathogens from the host and the IL-6–induced dominance of Th17 cells over Tregs may account for the disruption of the immune tolerance that is involved in the development of autoimmune and inflammatory diseases. The use of TCZ may be of interest to restore the immune balance between Th17 and Treg, and to avoid excessive immune stimulation.

A randomized controlled trial that included 30 kidney transplant patients with stable graft function, with subclinical inflammation defined on a routine biopsy as moderate interstitial inflammation (Banff classification i or ti 1–2 and t0) was performed by Chandran et al. (36). Subjects were randomized between a treatment group (TCZ 8 mg/kg monthly, six injections) and a placebo group. The authors showed a significant decrease in interstitial inflammation associated with an increased level of Tregs in 62.5% of subjects treated with TCZ versus 21.4% in the control group (*p* = 0.03). This suggest that using TCZ in the early stages of graft inflammation, and even before the rejection stage, could prevent long term graft damage.

It has been shown that ABMR is partly mediated by complement activation and antibody-dependent cellular cytotoxicity, mostly induced by IgG1 and 1Gg3 subclasses DSAs. A high risk of renal- and liver-allograft failure with anti-HLA IgG3 DSAs has been reported (37, 38). High levels of IgG3 DSAs were found mostly in kidney transplant patients who experienced acute rejection compared to patients without (39), and IgG3 and C1q^+^ iDSAs seemed to have the strongest association with allograft failure (40). That is why Shin et al. (41) performed, in 2020, an open-label study based on the 36 patients within Choi et al. cohort (24). Of the 36 patients, 12 were enrolled as a control group, while 14 patients with a DSA positive biopsy-proven cABMR, and with or without transplant glomerulopathy, received the SOC. The objective was to assess the levels of total IgG, anti-HLA-IgG and their subclasses at pre- and post-treatment and the possible involvement of specific IgG subclasses supression. The total IgG (12.2 ± 1.4 vs. 11.1 ± 1.6 g/L, *p*=0.09), IgG1 (9.3 ± 1.4 vs. 8.2 ± 1.6 g/L, *p*=0.08), IgG2 (3.7 ± 2.0 vs. 2.6 ± 0.8 g/L, *p*=0.09), and IgG3 (1.5 ± 0.9 vs. 0.8 ± 0.5 g/L, *p*=0.007) levels significantly or nearly significantly decreased at post-TCZ, whereas there was no significant reduction at post-treatment in the control group. This suggests that TCZ reduced the levels of major IgG components, IgG1, IgG2 and IgG3, resulting in total IgG reduction and, thus, this suppression by TCZ is unlikely to be subclass-specific. IgG4 levels did not change at post-TCZ, and the control group showed a significant reduction at post-treatment (0.4 ± 0.2 vs. 0.2 ± 0.1 g/L, *p*=0.03), probably linked to rituximab treatment. This shows that TCZ suppresses Ig-producing B cells including anti-HLA-IgG-producing B cells, and possibly including plasmablast and plasma cells, through inhibition of IL-6, a B cell, and plasmablast growth factor. This might be one of the factors contributing to the beneficial effect of TCZ observed in cABMR patients.

Clazakizumab is a humanized monoclonal IgG1 antibody with high affinity for IL-6 and a long half-life of approximately 30 days compared to TCZ, which is shorter, dependent on the concentration. This antibody has been systematically evaluated to treat rheumatoid and psoriatic arthritis in clinical trials, but has not yet been approved for clinical use. An investigator-driven, randomized, double-blind, placebo-controlled, parallel-group phase II pilot trial was conducted and published in 2021 (18). In this study, 20 kidney-transplant recipients with DSA-positive ABMR after a median of 10.6 years post-transplantation, were randomly assigned to receive clazakizumab or a placebo to assess safety, tolerability, and efficacy of the molecule. The main adverse events were infections and gastrointestinal illness, such as diverticulitis, leading to dose-diminution of clazakizumab. Within 12 weeks, clazakizumab decreased DSA MFI of 77%, but there were no significant difference in ABMR and TCMR incidence between the two groups. The key results of the secondary endpoint analysis were a slowed decline in eGFR and, after extended treatment, modulation of rejection-associated gene expression patterns, reduction of C4d scores, and, in some patients, resolution of ABMR activity. These results are promising, but further studies are needed to assess the safety and the incidence of adverse events such as diverticulitis.

Another relevant point that was raised by Choi et al. (24) is a potential rebound after stopping TCZ leading to rejection and suggesting accumulation of IL-6 under TCZ due to receptor blockage. Moreover, TCZ discontinuation act tardively in the immune response against the graft, i.e. at the time of antibody formation. Indeed, as shown in most of the studies presented in this review, TCZ, by blocking the IL-6R and the B cells in their production of antibodies, decreases the level of DSAs without making them disappear. However, we can imagine that when TCZ is stopped, the accumulation of IL-6 and the possibility for this cytokine to bind again to its receptor leads to a significant production of antibodies and therefore, to subsequently worsen humoral rejection. In that case, it may be interesting to introduce the TCZ before the creation of DSAs. Moreover, it would be interesting to add the rituximab, an anti-CD20, or another treatment that blocks B cells, in association with TCZ, to target both the causes and consequences. Even if the rituximab does not target directly the plasma cells (that does not express CD20), its effect on antibody reduction is described in many auto-immune diseases and also in the setting of HLA antibodies in desensitization studies (42).

Immunosuppressive treatment associated with TCZ should be explored to treat caABMR but also for maintenance treatment after kidney transplantation. In the study of Noble et al. (29), one patient received belatacept as a maintenance treatment before rejection and 18 patients were converted to belatacept after ABMR diagnostic. Belatacept is known to be responsible of more acute TCMR but is also associated with a better graft survival and renal function as compared to anti calcineurin drugs. The adjunction of TCZ to Belatacept may be an interesting approach.

Finally, TCZ has also been used in desensitization protocols in highly sensitized kidney-transplant candidates (43). A decrease in the level of anti-HLA antibodies and the number of plasma cells producing these antibodies in the bone marrow and spleen with TCZ has been shown in mice. In a phase I/II trial (23), TCZ was associated with IVIg in highly sensitized patients with failure to desensitize using standard protocols. The safety profile for treatment was favorable, and 5/10 patients were subsequently able to receive a transplant. Mean time to transplantation from first desensitization was 25 ± 10.5 months, but was significantly reduced to 8.1 ± 5.4 months, after TCZ. There was no ABMR on a routine biopsy conducted at 6 months, and no development of DSAs.

## Conclusion

Tocilizumab may be an alternative to SOC therapy in DSA positive caABMR or aABMR in kidney transplant recipients, either as a first-line treatment or after failure of SOC therapy. TCZ seems to reduce DSA levels and decrease kidney inflammation and microvascular lesions and seems to have a protective effect on renal function. Further studies are important to support and explore these results, in particular randomized and controlled studies.

## Author Contributions

JN, PM, and LR designed the study. LC made the literature search. LC and JN wrote the paper. TJ, BJ, and LR edited the manuscript. DG reviewed the kidney allograft biopsies from our patients. All authors contributed to the article and approved the submitted version.

## Conflict of Interest

The authors declare that the research was conducted in the absence of any commercial or financial relationships that could be construed as a potential conflict of interest.

## Publisher’s Note

All claims expressed in this article are solely those of the authors and do not necessarily represent those of their affiliated organizations, or those of the publisher, the editors and the reviewers. Any product that may be evaluated in this article, or claim that may be made by its manufacturer, is not guaranteed or endorsed by the publisher.

## References

[1] Lv J-C Zhang L-X. Prevalence and Disease Burden of Chronic Kidney Disease. Adv Exp Med Biol (2019) 1165:3−15. doi: 10.1007/978-981-13-8871-2_1 31399958

[2] Hill NR Fatoba ST Oke JL Hirst JA O’Callaghan CA Lasserson DS. Global Prevalence of Chronic Kidney Disease - A Systematic Review and Meta-Analysis. PloS One (2016) 11:e0158765. doi: 10.1371/journal.pone.0158765 27383068 PMC4934905

[3] Liyanage T Ninomiya T Jha V Neal B Patrice HM Okpechi I. Worldwide Access to Treatment for End-Stage Kidney Disease: A Systematic Review. Lancet (2015) 385:1975−82. doi: 10.1016/S0140-6736(14)61601-9 25777665

[4] Wolfe RA Ashby VB Milford EL Ojo AO Ettenger RE Agodoa LY. Comparison of Mortality in All Patients on Dialysis, Patients on Dialysis Awaiting Transplantation, and Recipients of a First Cadaveric Transplant. N Engl J Med (1999) 341:1725−30. doi: 10.1056/NEJM199912023412303 10580071

[5] Laupacis A Keown P Pus N Krueger H Ferguson B Wong C. A Study of the Quality of Life and Cost-Utility of Renal Transplantation. Kidney Int (1996) 50:235−42. doi: 10.1038/ki.1996.307 8807593

[6] Augustine J. Kidney Transplant: New Opportunities and Challenges. Cleve Clin J Med (2018) 85:138−44. doi: 10.3949/ccjm.85gr.18001 29425089

[7] Montgomery RA Loupy A Segev DL. Antibody-Mediated Rejection: New Approaches in Prevention and Management. Am J Transpl (2018) 18:3−17. doi: 10.1111/ajt.14584 29292861

[8] Chehade H Pascual M. The Challenge of Acute Antibody-Mediated Rejection in Kidney Transplantation. Transplantation (2016) 100:264−5. doi: 10.1097/TP.0000000000000959 26479286

[9] Mayrdorfer M Liefeldt L Wu K Rudolph B Zhang Q Friedersdorff F. Exploring the Complexity of Death-Censored Kidney Allograft Failure. J Am Soc Nephrol (2021) 32:1513−26. doi: 10.1681/ASN.2020081215 33883251 PMC8259637

[10] Sellarés J Reeve J Loupy A Mengel M Sis B Skene A. Molecular Diagnosis of Antibody-Mediated Rejection in Human Kidney Transplants. Am J Transplant (2013) 13:971−83. doi: 10.1111/ajt.12150 23414212

[11] Montgomery RA Zachary AA Racusen LC Leffell MS King KE Burdick J. Plasmapheresis and Intravenous Immune Globulin Provides Effective Rescue Therapy for Refractory Humoral Rejection and Allows Kidneys to be Successfully Transplanted Into Cross-Match-Positive Recipients. Transplantation (2000) 70:887−95. doi: 10.1097/00007890-200009270-00006 11014642

[12] Böhmig GA Wahrmann M Regele H Exner M Robl B Derfler K. Immunoadsorption in Severe C4d-Positive Acute Kidney Allograft Rejection: A Randomized Controlled Trial. Am Soc Transplant (2007) 7:117−21. doi: 10.1111/j.1600-6143.2006.01613.x 17109725

[13] Wan SS Ying TD Wyburn K Roberts DM Wyld M Chadban SJ. The Treatment of Antibody-Mediated Rejection in Kidney Transplantation: An Updated Systematic Review and Meta-Analysis. Transplantation (2018) 102:557−68. doi: 10.1097/TP.0000000000002049 29315141

[14] Sautenet B Blancho G Büchler M Morelon E Toupance O Barrou B. One-Year Results of the Effects of Rituximab on Acute Antibody-Mediated Rejection in Renal Transplantation: RITUX ERAH, a Multicenter Double-Blind Randomized Placebo-Controlled Trial. Transplantation (2016) 100:391−9. doi: 10.1097/TP.0000000000000958 26555944

[15] Kidney Disease: Improving Global Outcomes (KDIGO) Transplant Work Group. KDIGO Clinical Practice Guideline for the Care of Kidney Transplant Recipients. Am J Transplant (2009) 9:S1–155. doi: 10.1111/j.1600-6143.2009.02834.x 19845597

[16] Loupy A Haas M Roufosse C Naesens M Adam B Afrouzian M. The Banff 2019 Kidney Meeting Report (I): Updates on and Clarification of Criteria for T Cell- and Antibody-Mediated Rejection. Am J Transplant (2020) 20:2318−31. doi: 10.1111/ajt.15898 32463180 PMC7496245

[17] Eskandary F Regele H Baumann L Bond G Kozakowski N Wahrmann M. A Randomized Trial of Bortezomib in Late Antibody-Mediated Kidney Transplant Rejection. J Am Soc Nephrol (2018) 29:591−605. doi: 10.1681/ASN.2017070818 29242250 PMC5791086

[18] Doberer K Duerr M Halloran PF Eskandary F Budde K Regele H. A Randomized Clinical Trial of Anti-IL-6 Antibody Clazakizumab in Late Antibody-Mediated Kidney Transplant Rejection. J Am Soc Nephrol (2021) 32:708−22. doi: 10.1681/ASN.2020071106 33443079 PMC7920172

[19] Tanaka T Kishimoto T. The Biology and Medical Implications of Interleukin-6. Cancer Immunol Res (2014) 2:288−94. doi: 10.1158/2326-6066.CIR-14-0022 24764575

[20] Kim I Wu G Chai N Klein AS Jordan S. Anti-Interleukin 6 Receptor Antibodies Attenuate Antibody Recall Responses in a Mouse Model of Allosensitization. Transplantation (2014) 98:1262−70. doi: 10.1097/TP.0000000000000437 25286051

[21] Jordan SC Choi J Kim I Wu G Toyoda M Shin B. Interleukin-6, A Cytokine Critical to Mediation of Inflammation, Autoimmunity and Allograft Rejection: Therapeutic Implications of IL-6 Receptor Blockade. Transplantation (2017) 101:32−44. doi: 10.1097/TP.0000000000001452 27547870

[22] Nishimoto N Terao K Mima T Nakahara H Takagi N Kakehi T. Mechanisms and Pathologic Significances in Increase in Serum Interleukin-6 (IL-6) and Soluble IL-6 Receptor After Administration of an Anti-IL-6 Receptor Antibody, Tocilizumab, in Patients With Rheumatoid Arthritis and Castleman Disease. Blood (2008) 112:3959−64. doi: 10.1182/blood-2008-05-155846 18784373

[23] Vo AA Choi J Kim I Louie S Cisneros K Kahwaji J. A Phase I/II Trial of the Interleukin-6 Receptor-Specific Humanized Monoclonal (Tocilizumab) + Intravenous Immunoglobulin in Difficult to Desensitize Patients. Transplantation (2015) 99:2356−63. doi: 10.1097/TP.0000000000000741 26018350

[24] Choi J Aubert O Vo A Loupy A Haas M Puliyanda D. Assessment of Tocilizumab (Anti-Interleukin-6 Receptor Monoclonal) as a Potential Treatment for Chronic Antibody-Mediated Rejection and Transplant Glomerulopathy in HLA-Sensitized Renal Allograft Recipients. Am J Transplant (2017) 17:2381−9. doi: 10.1111/ajt.14228 28199785

[25] Massat M Congy-Jolivet N Hebral A-L Esposito L Marion O Delas A. Do Anti-IL-6R Blockers Have a Beneficial Effect in the Treatment of Antibody-Mediated Rejection Resistant to Standard Therapy After Kidney Transplantation? Am J Transplant (2021) 21:1641−9. doi: 10.1111/ajt.16391 33141487

[26] Lavacca A Presta R Gai C Mella A Gallo E Camussi G. Early Effects of First-Line Treatment With Anti-Interleukin-6 Receptor Antibody Tocilizumab for Chronic Active Antibody-Mediated Rejection in Kidney Transplantation. Clin Transplant (2020) 34:e13908. doi: 10.1111/ctr.13908 32415711

[27] Pottebaum AA Venkatachalam K Liu C Brennan DC Murad H Malone AF. Efficacy and Safety of Tocilizumab in the Treatment of Acute Active Antibody-Mediated Rejection in Kidney Transplant Recipients. Transplant Direct (2020) 6:e543. doi: 10.1097/TXD.0000000000000988 32309629 PMC7145000

[28] Kumar D Yakubu I Safavi F Levy M Moinuddin I Kimball P. Lack of Histological and Molecular Signature Response to Tocilizumab in Kidney Transplants With Chronic Active Antibody Mediated Rejection: A Case Series. Kidney (2020) 360 1:663–70. doi: 10.34067/KID.0000182019 PMC8815553 35372943

[29] Noble J Giovaninni D Laamech R. Tocilizumab in the Treatment of Chronic Antibody-Mediated Rejection Post Kidney Transplantation: Clinical and Histological Monitoring. Front Med (Lausanne) (2021) 8:790547. doi: 10.3389/fmed.2021.790547 35004757 PMC8739887

[30] De Serres SA Noël R Côté I Lapointe I Wagner E Riopel J. 2013 Banff Criteria for Chronic Active Antibody-Mediated Rejection: Assessment in a Real-Life Setting. Am J Transplant (2016) 16:1516−25. doi: 10.1111/ajt.13624 26602055

[31] Yamada C Ramon DS Cascalho M Sung RS Leichtman AB Samaniego M. Efficacy of Plasmapheresis on Donor-Specific Antibody Reduction by HLA Specificity in Post-Kidney Transplant Recipients. Transfusion (Paris) (2015) 5:727−35. doi: 10.1111/trf.12923 PMC4911015 25385678

[32] Scott LJ. Tocilizumab: A Review in Rheumatoid Arthritis. Drugs (2017) 77:1865−79. doi: 10.1007/s40265-017-0829-7 29094311 PMC5736769

[33] Haraoui B Casado G Czirják L Taylor A Bernasconi C Reiss W. Patterns of Tocilizumab Use, Effectiveness and Safety in Patients With Rheumatoid Arthritis: Core Data Results From a Set of Multinational Observational Studies. Clin Exp Rheumatol (2017) 35:899−906. 28516886

[34] Morel J Constantin A Baron G Dernis E Flipo RM Rist S. Risk Factors of Serious Infections in Patients With Rheumatoid Arthritis Treated With Tocilizumab in the French Registry REGATE. Rheumatology (2017) 56:1746−54. doi: 10.1093/rheumatology/kex238 28957557

[35] Khanna D Denton CP Jahreis A van Laar JM Frech TM Anderson ME. Safety and Efficacy of Subcutaneous Tocilizumab in Adults With Systemic Sclerosis (Fasscinate): A Phase 2, Randomised, Controlled Trial. Lancet (2016) 387:2630−40. doi: 10.1016/S0140-6736(16)00232-4 27156934

[36] Chandran S Leung J Hu C Laszik ZG Tang Q Vincenti FG. Interleukin-6 Blockade With Tocilizumab Increases Tregs and Reduces T Effector Cytokines in Renal Graft Inflammation: A Randomized Controlled Trial. Am J Transpl (2021) 21:2543−54. doi: 10.1111/ajt.16459 33331082

[37] Everly MJ Rebellato LM Haisch CE Briley KP Bolin P Kendrick WT. Impact of IgM and IgG3 Anti-HLA Alloantibodies in Primary Renal Allograft Recipients. Transplantation (2014) 97:494−501. doi: 10.1097/01.TP.0000441362.11232.48 24487396

[38] O’Leary JG Kaneku H Banuelos N Jennings LW Klintmalm GB Terasaki PI. Impact of IgG3 Subclass and C1q-Fixing Donor-Specific HLA Alloantibodies on Rejection and Survival in Liver Transplantation. Am J Transpl (2015) 15:1003−13. doi: 10.1111/ajt.13153 25772599

[39] Gao Z McAlister VC Wright JR McAlister CC Peltekian K MacDonald AS. Immunoglobulin-G Subclass Antidonor Reactivity in Transplant Recipients. Liver Transplant (2004) 10:1055−9. doi: 10.1002/lt.20154 15390333

[40] Lefaucheur C Viglietti D Bentlejewski C Duong van Huyen J-P Vernerey D Aubert O. IgG Donor-Specific Anti-Human HLA Antibody Subclasses and Kidney Allograft Antibody-Mediated Injury. J Am Soc Nephrol (2016) 27:293−304. doi: 10.1681/ASN.2014111120 26293822 PMC4696574

[41] Shin B-H Everly MJ Zhang H Choi J Vo A Zhang X. Impact of Tocilizumab (Anti-IL-6r) Treatment on Immunoglobulins and Anti-HLA Antibodies in Kidney Transplant Patients With Chronic Antibody-Mediated Rejection. Transplantation (2020) 104:856−63. doi: 10.1097/TP.0000000000002895 31385933

[42] Vo AA Choi J Cisneros K Reinsmoen N Haas M Ge S. Benefits of Rituximab Combined With Intravenous Immunoglobulin for Desensitization in Kidney Transplant Recipients. Transplantation (2014) 98:312. doi: 10.1097/TP.0000000000000064 24770617

[43] Weinhard J Noble J Jouve T Malvezzi P Rostaing L. Tocilizumab and Desensitization in Kidney Transplant Candidates: Personal Experience and Literature Review. J Clin Med (2021) 10:4359. doi: 10.3390/jcm10194359 34640377 PMC8509506

